# Highly Efficient Liquid-Phase Hydrogenation of Naringin Using a Recyclable Pd/C Catalyst

**DOI:** 10.3390/ma12010046

**Published:** 2018-12-24

**Authors:** Jiamin Zhao, Ying Yuan, Xiuhong Meng, Linhai Duan, Rujin Zhou

**Affiliations:** Department of Chemical Engineering, Guangdong University of Petrochemical Technology, Maoming 525000, China; zhaojiamin08031223@163.com (J.Z.); yuanyingsdlc@163.com (Y.Y.); mengxiuh@163.com (X.M.)

**Keywords:** Pd/C, Catalyst, Hydrogenation, Naringin, Dihydrochalcone

## Abstract

A highly efficient liquid-phase hydrogenation reaction using a recyclable palladium on carbon (Pd/C) catalyst has been used for the transformation of naringin to its corresponding dihydrochalcone. The effects of various solvents on the hydrogenation process were studied, with water being identified as the optimal solvent. The analysis also revealed that sodium hydroxide (NaOH) can accumulate on the surface of the Pd/C catalyst in alcoholic solvents, leading to its inactivation. The higher solubility of NaOH in water implies that it remains in solution and does not accumulate on the Pd/C catalyst surface, ensuring the catalytic activity and stability.

## 1. Introduction

There has been significant interest in dihydrochalcones (DHC) since Horwits reported their strong sweetness in 1963 [1]; these heterocycles are also known to exhibit antioxidant or antidiabetic activities [2,3]. For example, Chao et al. [4] used phlorizin as a DHC template to develop three antidiabetic drugs approved by the Food and Drug Administration (FDA) and the European Medicine Agency (EMA) [5]. Snijman et al. [6] reported that the dihydrochalcones aspalathin and nothofagin exhibit strong antioxidant activities, implying that they are ingested into the human body as intact glycosides. Janvier et al. [7] gained approval for using the dihydrochalcone neohesperidin as a food additive (E959) in Europe. Naringin is a flavanone occurring as the major component of exocarpium and pomelo, which are cultivated widely in south China [8,9,10]. Therefore, processes to convert naringin to value-added products on an industrial scale must be developed.

**Scheme 1:**
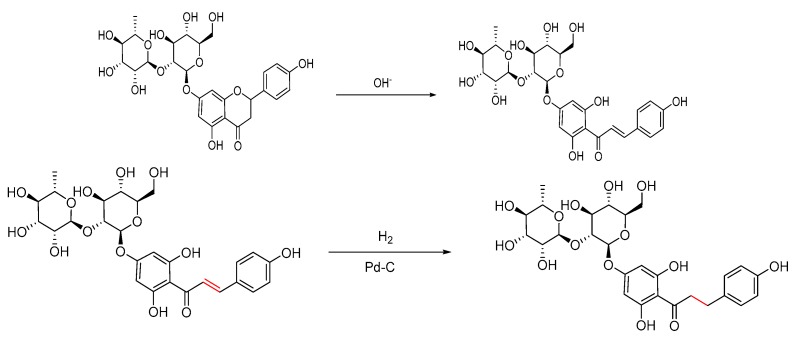
The reaction scheme of hydrogenation of naringin.

Naringin dihydrochalcone (NDC) can be prepared from naringin via a two-step alkali- mediated hydrogenation reaction at high pressures [11]. First, NaOH(aq) or (KOH(aq)) is used as a base for ring-opening of the tetrahydropyran-4-one fragment of naringin to afford the corresponding chalcone intermediate with an (E)-alkene bond. Second, selective hydrogenation of this alkene bond using Pd/C (or Raney nickel) catalyst at high hydrogen pressures yield the dihydrochalcone product; the reaction scheme is shown in Scheme 1. The key feature of this reaction is the selective hydrogenation of the C=C bond of the DHC intermediate over its C=O functionality. An ideal process would involve hydrogenation at medium-to-low hydrogen pressures at an ambient temperature using a heterogeneous catalyst that can be easily recovered and recycled [12,13]. Pd/C has often been used as a catalyst to selectively reduce the C=C bond of α, β-unsaturated carbonyl compounds, and the efficacy and mechanism of this reaction has been proven by experimental and theoretical results [14,15,16] and studies [17,18,19].

The choice of an appropriate solvent is critical for the success of hydrogenation using heterogeneous catalysis; the physical properties of solvents can significantly influence the yield and distribution of the hydrogenated products. An ideal solvent in a catalytic reaction can dissolve the solid reactants and products effectively, enhance conversion rates, and ensure that the catalyst surface remains free of inhibitors [20]. Alcohols (e.g., methanol or ethanol) are the most widely used solvents for reported heterogeneous catalytic hydrogenation reactions [21]. Wei et al. [22] have shown that Pd catalysts exhibit higher activity for hydrogenation reactions in toluene and ethanol, than in DMF, acetonitrile, or water. However, Ma et al. [23,24] reported that water was a more effective solvent for liquid-phase hydrodechlorination of chlorophenols catalyzed by Raney nickel. However, there has been little research on the effects of different solvents on liquid-phase catalytic hydrogenation of naringin.

In this study, our primary aim was to develop a practical process for catalytic hydrogenation of naringin to afford naringin dihydrochalcone using Pd/C as a recyclable heterogeneous catalyst. This was achieved by studying the effects of different solvents and the influence of water content on the yield of hydrogenation. The effects of solvents and reaction conditions on the structure of the recovered Pd/C catalyst was also studied using transmission electron microscopy (TEM), X-ray photoelectron spectroscopy (XPS), etc.

## 2. Experimental Section

### 2.1. Materials and Methods

The 732 cation exchange resin was purchased from Shape Chemical, Shanghai, China. Naringin was obtained from the National Institute for the Control of Pharmaceutical and Biological Products in Beijing, China. Other chemicals of analytical grade were purchased from Sigma-Aldrich, St. Louis, MO, USA.

TEM characterization was performed using a Hitachi HT-7700 microscope (Hitachi Corporation, Tokyo, Japan). High-resolution TEM (HRTEM) was performed using a Tecnai G2 F30 S-Twin microscope (Thermo Fisher Scientific, MA, USA) operated at an acceleration voltage of 300 kV.

The surface composition of the catalyst was analyzed using XPS (Thermo Escalab 250Xi XPS) (Thermo Fisher Scientific, MA, USA) with Al Kα radiation, operated at 15 kV and 14.9 mA, as the excitation source. Binding energy values were referenced to the C (1s) peak at 285.0 eV.

### 2.2. Preparation of Pd/C Catalyst

The 10 wt % Pd/C catalyst was prepared by adding 0.2 g of activated carbon to 40 mL of water containing 0.11 g Na_2_PdCl_4_ (Sigma-Aldrich, St. Louis, MO, USA). After sonication for 10 min and stirring for 4 h, NH_3_·H_2_O (Sigma-Aldrich, St. Louis, MO, USA) was added dropwise to the solution for adjusting pH to 8.5. Next, the Pd adsorbed on activated carbon was reduced by adding 10 mL of freshly prepared 0.08 mol L-1 NaBH_4_ (Sigma-Aldrich, St. Louis, MO, USA) solution. The mixture was further stirred for 12 h. Finally, the Pd/C was washed and collected by several rounds of centrifugation and the final product was dried under vacuum for 12 h.

### 2.3. Procedures Used to Carry Out Hydrogenation Reactions

Liquid-phase hydrogenation was performed in a 250-mL Hastelloy autoclave (Parr, Illinois, USA) fitted with a mechanical stirrer and an electric temperature controller. In a typical reaction, the reactor was loaded with 0.50 g of the reduced heterogeneous Pd/C catalyst and 150 mL of an aqueous solution containing 5.0 g of naringin and 0.6 g of NaOH (Sigma-Aldrich, St. Louis, MO, USA).

The reactor was purged with H_2_ to remove air and was then pressurized to 1.5 MPa using H_2_. The reactor was then heated to 40 °C and stirred at 600 rpm for 10–14 h. The reaction mixture was then filtered to recover the heterogeneous catalyst and the filtrate was passed through a type 732 cation exchanger (Shape Chemical, Shanghai, China) (to exchange Na^+^ for H^+^ cations). This resulted in crystallization of the dihydrochalcone product from the solution, and the crystals were collected using vacuum filtration [8].

## 3. Results and Discussion

### 3.1. Characterization of Pd/C and the Product DHC

**Figure 1:**
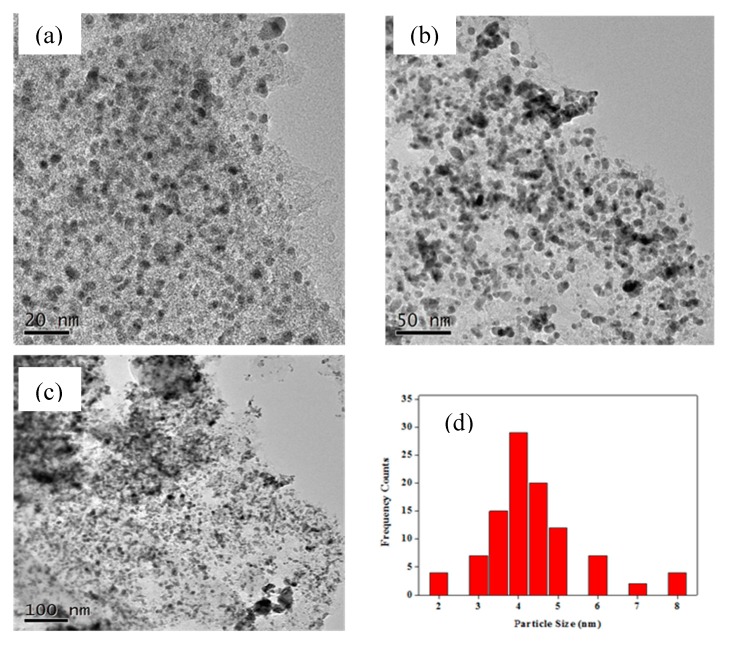
Transmission electron microscopy (TEM) images of 10 wt % Pd/C with different magnifications (**a**–**c**) and size distribution of Pd particles (**d**).

**Figure 2:**
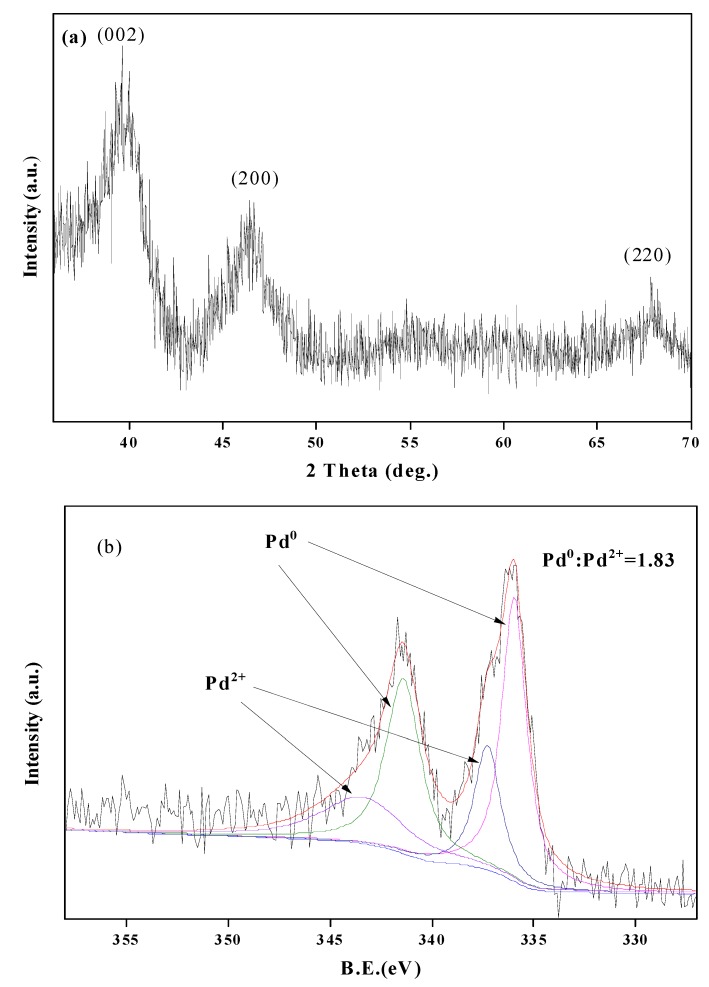
(**a**) XRD and (**b**) X-ray photoelectron spectroscopy (XPS) patterns of Pd/C.

The TEM images (Figure 1) of the as-synthesized Pd/C indicated that Pd nanoparticles with diameters of 4.3 nm are dispersed uniformly on the carbon black. The X-ray diffraction (XRD) patterns of the Pd/C sample (Figure 2a) exhibit three typical peaks at 2θ = 40.1°, 47.4°, and 68.7°, corresponding to the (111), (200), and (220) reflections, respectively, of crystalline Pd, which matches well with PDF 65-6174. The diameters of the Pd nanoparticles can also be measured by XRD using the Scherrer equation [25]: L=Kλ/βcosθ. Where λ is the X-ray wavelength in nanometer (nm), β is the peak width of the diffraction peak profile at half maximum height resulting from small crystallite size in radians and K is a constant related to crystallite shape, normally taken as 0.89. The diameter of the Pd nanoparticle calculated using Scherrer equation is 0.41 nm, which is similar to the average particle size (0.43 nm) observed from TEM. The XPS spectrum of Pd/C (Figure 2b) shows two symmetrical peaks assigned to the Pd 3d 5/2 and Pd 3d 3/2 core levels. The peaks at 335.9 and 341.4 eV are attributed to metallic Pd0, while those at 337.3 eV and 343.4 eV correspond to the Pd 2+ species. The percentage of metallic Pd0 was calculated from the relative areas under these peaks; metallic Pd0 was found to be the main metal species on the surface of the as-prepared catalyst (65 wt %).

**Figure 3:**
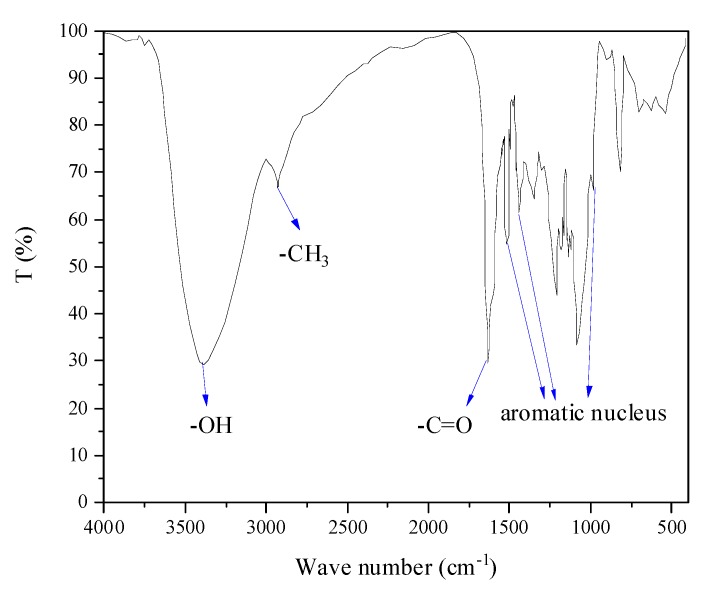
IR absorption of naringin hydrochalcone (DHC).

The product naringin hydrochalcone was characterized by infrared spectroscopy (IR) absorption (Figure 3). IR absorption at 3390 cm^−1^ (-OH), 2923.84 cm^−1^ (-CH_3_), 1631 cm^−1^ (conjugated -C=O), 1513 cm^−1^, 1438 cm^−1^ and 817 cm^−1^ (aromatic nucleus) were indicative of a hydroxylated dihydrochalcone, similar to the result of Tang et al. [8].

### 3.2. Solvent Effect on the Hydrogenation Reaction of Naringin over Pd/C Catalyst

**Figure 4:**
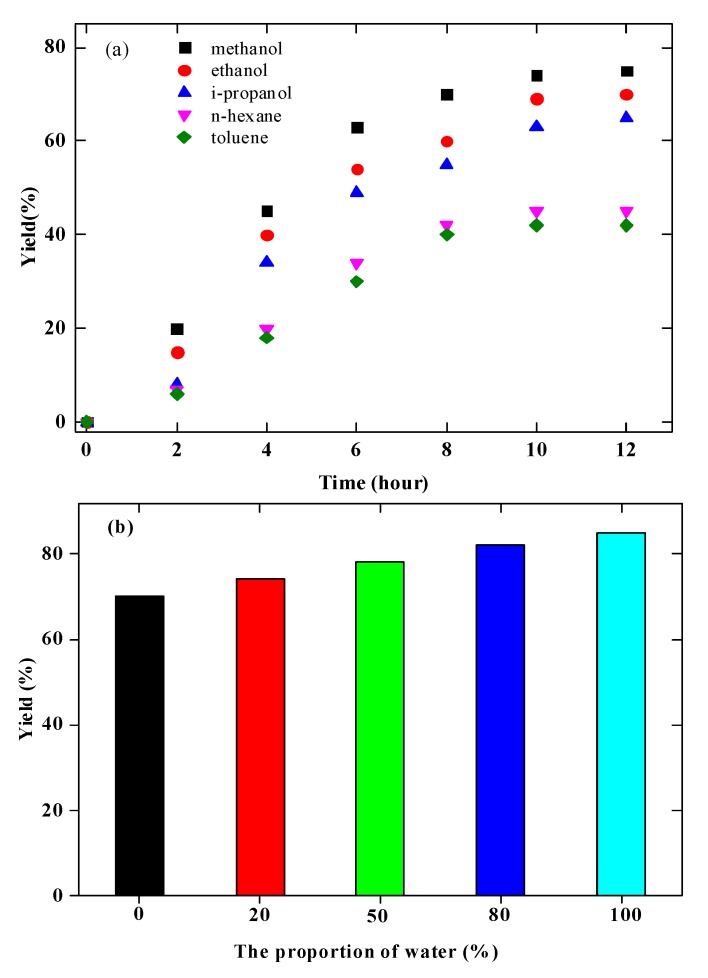
Hydrogenation of naringin in different solvents over 10 wt % Pd/C catalyst. (**a**) Hydrogenation of naringin in various solvents. (**b**) Hydrogenation of naringin in various ethanol–water mixtures. Reaction conditions: 150 mL of each solvent, 5 g of naringin, solvent pH 12, 0.5 g of 10 wt % Pd/C(0), temperature of 45 °C, and H_2_ pressure of 1.5 MPa.

The hydrogenation of naringin over Pd/C catalyst was examined in various solvents, including methanol, ethanol, iso-propanol, and n-hexane (Figure 4a). It was found that naringin could be hydrogenated in all these solvents, with DHC yields depending strongly on the solvent, according to the following order: methanol > ethanol > iso-propanol > n-hexane > toluene. The Pd/C catalyst showed greater activity in alcoholic solvents than in n-hexane and toluene. Therefore, protic solvents are better solvents than aprotic ones for liquid-phase hydrogenation reactions of naringin. These results are consistent with previous reports, which describe that hydrogenation of non-polar substrates in polar solvents give the best yields of hydrogenated products [20,26]. The rates of these hydrogenation reactions were also dependent on solvent polarity, with hydrogenation reactions occurring faster in higher-polarity solvents. Indeed, for alcoholic solvents, DHC yield was directly proportional to the order of the normalized empirical parameter (ETN), dielectric constant (ε), and dipole moment (μ) of the solvents [23,27,28].

Water, a highly polar protic solvent, can be used as an additive to increase the polarity of alcoholic solvents. Therefore, a series of mixed alcohol/water solvents were investigated to determine the impact of solvent polarity on hydrogenation reactions. A series of Pd/C-catalyzed hydrogenation reactions of naringin were performed in different mixed solvents, i.e., ethanol, 20 wt % water-ethanol (20/80, *v*/*v*), 50 wt % water-ethanol (50/50, *v*/*v*), 80 wt % water-ethanol (80/20, *v*/*v*), and water (Figure 4b). These experiments revealed that the highest DHC yield was obtained using water as the solvent, followed by progressively lower DHC yields for 80 wt % water-ethanol, 50 wt % water-ethanol, 20 wt % water–ethanol, and ethanol. These results showed that the rate of hydrogenation increased with increasing polarity of the mixed ethanol-water solvents. Therefore, the rate of hydrogenation of naringin over the Pd/C catalyst can be increased significantly by simply adding water to the alcoholic solvent.

**Figure 5:**
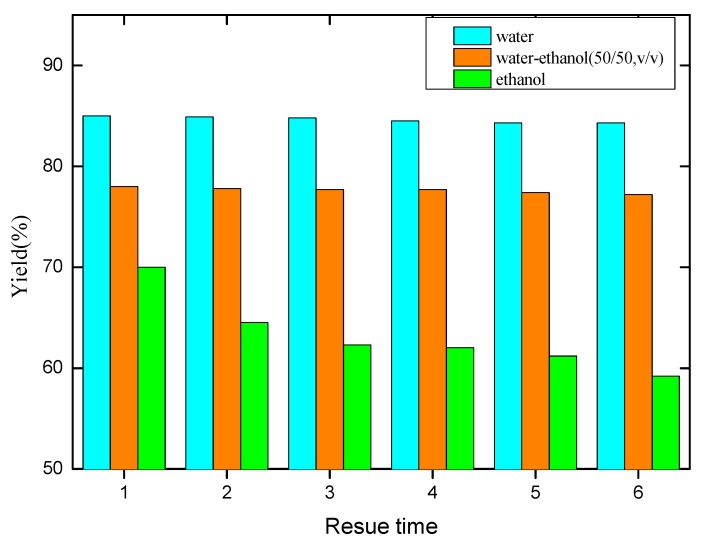
Repeated hydrogenation of naringin in water, water-ethanol, and ethanol.

The stability and recyclability of the 10 wt % Pd/C catalyst used for hydrogenation in water, 50 wt % water-ethanol (50/50, *v*/*v*), and ethanol were then investigated (Figure 5). In ethanol, the initial DHC yield was 70 wt % after 12 h; however, this yield dropped to 59 wt % on using Pd/C catalyst that had been recycled five times. However, the DHC yield obtained using water or water-ethanol (50/50, *v*/*v*) as solvent remained almost unchanged after six rounds of catalyst recycling, indicating superior stability of the Pd/C catalyst in these solvents. Therefore, we concluded that water was the best solvent for performing Pd/C-catalyzed liquid-phase hydrogenation of naringin.

### 3.3. The Influence of Solvent on Pd/C Catalyst Structure

**Figure 6:**
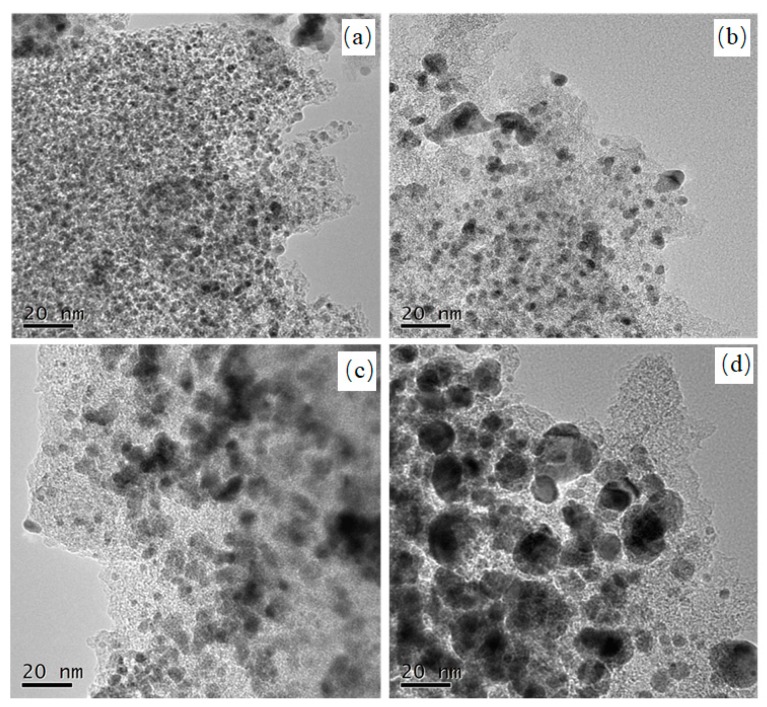
TEM images of the Pd/C catalysts fresh (**a**), after 6 cycles of hydrogenation in water (**b**), after 6 cycles of hydrogenation in ethanol-water (50/50) (**c**), after 6 cycles of hydrogenation in ethanol (**d**).

Having shown that among the solvents studied, water is the optimal solvent for hydrogenation of naringin [20,28,29,30], we used TEM and XPS studies to elucidate the solvent effect on the structure of the Pd/C catalyst. The recovered 10 wt % Pd/C catalyst after six rounds of use for hydrogenation of naringin was analyzed using TEM. The surface morphologies of 10 wt % Pd/C catalyst that had been reused six times using water (Figure 6b) and 50 wt % water-ethanol (Figure 6c) as solvent were almost identical to that of the original Pd/C catalyst. On the other hand, when ethanol was used as the solvent, the morphology of the recovered Pd/C catalyst changed significantly, with significant amounts of crystalline material deposited on its surface after six rounds of use in the hydrogenation reactions (Figure 6d).

To gain more information about the structural composition of this crystalline material, 10 wt % Pd/C catalysts recovered from ethanol–water (50/50) and ethanol reactions were subjected to XPS analysis. Table 1 provides the XPS analysis results of the composition of fresh and reused Pd/C catalysts. This table shows the presence of Pd3d and O1s emissions at 336.39 eV and 532.60 eV, respectively, probably due to the presence of PdO. The biggest difference between catalyst samples recovered from hydrogenation reactions in water and ethanol was in their Pd3d and Na1s emissions. The Pd-to-Na ratios of a Pd/C catalyst recovered from hydrogenation reactions in water and ethanol were 1.79:1 and 0.38:1, respectively. This indicates that the crystalline material observed on the surface of the catalyst recovered from hydrogenation reactions in ethanol is probably NaOH. Therefore, the reduced catalytic activity of Pd/C in ethanol is due to NaOH blocking the catalytically active sites on the catalyst surface, preventing it from effectively absorbing hydrogen and the naringin substrate. However, when water is present, its higher polarity ensures that NaOH remains in solution, preventing the Pd/C catalyst surface from becoming deactivated.

### 3.4. Optimal Reaction Conditions for Naringin Hydrogenation

**Figure 7:**
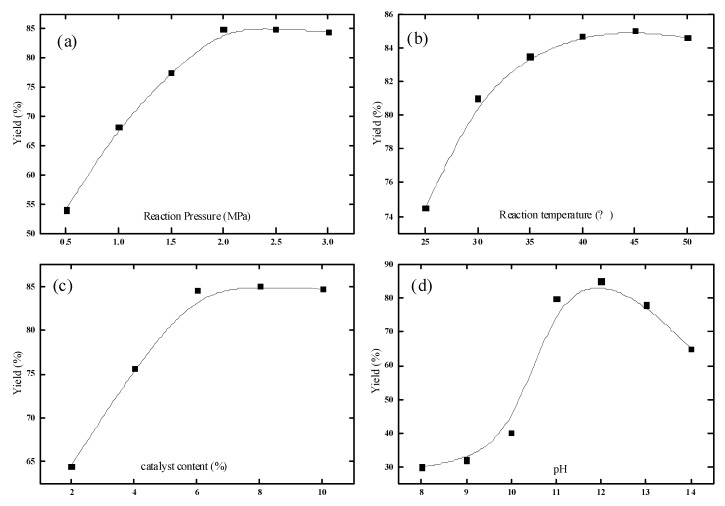
Effects of reaction pressure (**a**), temperature (**b**), catalyst content (**c**), and pH (**d**) on the DHC yield. The Reaction conditions: 150 mL of each solvent, 5 g of naringin, solvent pH 12, 0.5 g of 10 wt % Pd/C(0), temperature of 45 °C, and H_2_ pressure of 1.5 MPa.

The effects of reaction temperature, pressure, pH, and catalyst content on the DHC yield in hydrogenation of naringin were then investigated (Figure 7). Figure 7a shows that the DHC yield in these reactions increased up to a maximum at hydrogen pressure of 2.0 MPa, with no further increase in yield at higher pressures. Figure 7b shows that the optimal temperature for these hydrogenation reactions was 42–45 °C. Higher temperatures resulted in catalyst degradation, leading to lower DHC yields. Increasing the amount of catalyst from 2 wt % to 10 wt % significantly increased the DHC yield, while the optimum basicity of the NaOH(aq)solvent was found to be pH 11.8 (Figure 7d). The role of NaOH on the reaction is to open heterocycles of naringin for forming the corresponding chalcone intermediate. If the pH is lower than 11, the heterocycles cannot be opened. If the pH is too high, the glycoside bonds on naringin may be destroyed, and the corresponding chalcone intermediate cannot be formed. These results were combined to design the optimal conditions for this hydrogenation reaction: hydrogen reaction pressure of 2.0–2.5 MPa, reaction temperature of 40–45 °C, pH 11.5–12.0, and Pd/C catalyst loading of 8–10 mol wt %.

## 4. Conclusions

The effects of various solvent systems on the Pd/C-catalyzed liquid-phase hydrogenation of naringin have been investigated. Water was found to be the optimal solvent for affording the corresponding dihydrochalcone in good yield. The presence of water was critical for preventing accumulation of NaOH on the surface of the Pd/C catalyst, which can lead to catalyst deactivation in alcoholic solvents. An optimal high-yielding and scalable process was developed using a hydrogen pressure of 2.0–2.5 MPa, a reaction temperature of 40–45 °C, an aqueous solvent of pH 11.5–12.0, and a recyclable Pd/C catalyst loading of 8–10 wt %.

## Figures and Tables

**Table 1:** XPS analysis of fresh and recycled Pd/C catalysts.

Peak _ ID	Area		Centre		AT (wt %)	
	Ethanol-Water	Ethanol	Ethanol-Water	Ethanol	Ethanol-Water	Ethanol
O1s	18,667	12,383	532.60	532.42	83.4	83.63
Na1s	2700	3323	1071.52	1071.47	4.63	11.87
Pd3d	19,280	6107	336.39	336.11	11.96	4.5

## References

[1] Horowitz R.M., Gentili B. (1963). Flavonoids of citrus-VI. Tetrahedron.

[2] Eichenberger M., Lehka B.J., Folly C., Fischer D., Martens S., Simón E., Naesby M. (2017). Metabolic engineering of Saccharomyces cerevisiae for de novo production of dihydrochalcones with known antioxidant, antidiabetic, and sweet tasting properties. Metab. Eng..

[3] Gutmann A., Bungaruang L., Weber H., Leypold M., Breinbauer R., Nidetzky B. (2014). Towards the synthesis of glycosylated dihydrochalcone natural products using glycosyltransferase-catalysed cascade reactions. Green Chem..

[4] Chao E.C. (2014). SGLT-2 Inhibitors: A New Mechanism for Glycemic Control. Clin. Diabetes.

[5] Scheen A.J. (2015). Pharmacokinetics, pharmacodynamics and clinical use of SGLT2 inhibitors in patients with type 2 diabetes mellitus and chronic kidney disease. Clin. Pharmacokinet..

[6] Snijman P.W., Joubert E., Ferreira D., Li X.-C., Ding Y., Green I.R., Gelderblom W.C. (2009). Antioxidant activity of the dihydrochalcones aspalathin and nothofagin and their corresponding flavones in relation to other rooibos (*Aspalathus linearis*) flavonoids, epigallocatechin gallate, and Trolox. J. Agric. Food Chem..

[7] Janvier S., Goscinny S., Donne C.L., Loco J.V. (2015). Low-calorie sweeteners in food and food supplements on the Italian market. Food Addit. Contam. Part B.

[8] Tang D.-M., Zhu C.-F., Zhong S.-A., Zhou M.-D. (2011). Extraction of naringin from pomelo peels as dihydrochalcone’s precursor. J. Sep. Sci..

[9] Yuan W., Feng Y., Xie A., Zhang X., Huang F., Li S., Zhang X., Shen Y. (2016). Nitrogen-doped nanoporous carbon derived from waste pomelo peel as a metal-free electrocatalyst for the oxygen reduction reaction. Nanoscale.

[10] Huang R., Cao M., Guo H., Qi W., Su R., He Z. (2014). Enhanced ethanol production from pomelo peel waste by integrated hydrothermal treatment, multienzyme formulation, and fed-batch operation. J. Agric. Food Chem..

[11] Wild J., Huber U. (1976). Process for Making Neohesperidine Dihydrochalcone. U.S. Patent.

[12] Wang Y., Yao J., Li H., Su D., Antonietti M. (2011). Highly selective hydrogenation of phenol and derivatives over a Pd@ carbon nitride catalyst in aqueous media. J. Am. Chem. Soc..

[13] Khan F.-A., Vallat A., Süss-Fink G. (2012). Highly selective C C bond hydrogenation in α, β-unsaturated ketones catalyzed by hectorite-supported ruthenium nanoparticles. J. Mol. Catal. A Chem..

[14] Kantam L., Parsharamulu T., Manorama S. (2012). Layered double hydroxides supported nano palladium: An efficient catalyst for the chemoselective hydrogenation of olefinic bonds. J. Mol. Catal. A Chem..

[15] Zhao B.H., Chen J.G., Liu X., Liu Z.W., Hao Z., Xiao J., Liu Z.T. (2012). Selective hydrogenation of cinnamaldehyde over Pt and Pd supported on multiwalled carbon nanotubes in a CO_2_-expanded alcoholic medium. Ind. Eng. Chem. Res..

[16] Morimoto N., Yamamoto S.-I., Takeuchi Y., Nishina Y. (2013). Palladium on graphene: The in situ generation of a catalyst for the chemoselective reduction of α, β-unsaturated carbonyl compounds. RSC Adv..

[17] Delbecq F., Sautet P. (1995). Competitive C=C and C=O Adsorption of α-β-Unsaturated Aldehydes on Pt and Pd Surfaces in Relation with the Selectivity of Hydrogenation Reactions: A Theoretical Approach. J. Catal..

[18] Pallassana V., Neurock M. (1999). Theoretical density functional study of the hydrogenation of maleic acid over Pd and Re surfaces. Chem. Eng. Sci..

[19] Manyar H.G., Yang B., Daly H., Moor H., McMonagle S., Tao Y., Yadav G.D., Goguet A., Hu P., Hardacre C. (2013). Selective Hydrogenation of α, β-Unsaturated Aldehydes and Ketones using Novel Manganese Oxide and Platinum Supported on Manganese Oxide Octahedral Molecular Sieves as Catalysts. ChemCatChem.

[20] Mukherjee S., Vannice M.A. (2006). Solvent effects in liquid-phase reactions: I. Activity and selectivity during citral hydrogenation on Pt/SiO_2_ and evaluation of mass transfer effects. J. Catal..

[21] Gong Y., Zhang P., Xu X., Li Y., Li H., Wang Y. (2013). A novel catalyst Pd@ ompg-C_3_N_4_ for highly chemoselective hydrogenation of quinoline under mild conditions. J. Catal..

[22] Wei Z., Gong Y., Xiong T., Zhang P., Li H., Wang Y. (2015). Highly efficient and chemoselective hydrogenation of α, β-unsaturated carbonyls over Pd/N-doped hierarchically porous carbon. Catal. Sci. Technol..

[23] Ma X., Liu Y., Li X., Xu J., Gu G., Xia C. (2015). Water: The most effective solvent for liquid-phase hydrodechlorination of chlorophenols over Raney Ni catalyst. Appl. Catal. B Environ..

[24] Ma X., Liu Y., Liu S., Xia C. (2014). Water-promoted catalytic hydrodechlorination of transformer oil-contained PCBs in liquid system under mild conditions. Appl. Catal. B Environ..

[25] Monshi A., Foroughi M.R., Monshi M.R. (2012). Modified Scherrer equation to estimate more accurately nano-crystallite size using XRD. World J. Nano Sci. Eng..

[26] Rajadhyaksha R., Karwa S. (1986). Solvent effects in catalytic hydrogenation. Chem. Eng. Sci..

[27] Lide D., Haynes W., Lide D.R. (2009). CRC Handbook of Chemistry and Physics: A Ready-Reference Book of Chemical and Physical Data.

[28] Reichardt C., Welton T. (2011). Solvents and Solvent Effects in Organic Chemistry.

[29] Akpa B., D’Agostino C., Gladden L., Hindle K., Manyar H., McGregor J., Li R., Neurock M., Sinha N., Stitt E.H. (2012). Solvent effects in the hydrogenation of 2-butanone. J. Catal..

[30] Wan H., Vitter A., Chaudhari R.V., Subramaniam B. (2014). Kinetic investigations of unusual solvent effects during Ru/C catalyzed hydrogenation of model oxygenates. J. Catal..

